# Retrospective assessment of antimicrobial stewardship initiative in outpatient use of ertapenem for uncomplicated extended spectrum beta lactamase *Enterobacteriaceae* urinary tract infections

**DOI:** 10.1186/s12879-021-06458-0

**Published:** 2021-08-16

**Authors:** Carrie P. Wong, Thomas Delate, Elizabeth Hudson, Julia K. Nguyen, Su-Jau Yang, Mariana Abraham

**Affiliations:** 1 Kaiser Permanente Northern California Ambulatory Care Pharmacy, Kaiser Permanente Santa Clara Medical Center, 700 Lawrence Expy, Santa Clara, CA 95051 USA; 2 Pharmacy Outcomes Research Group, Kaiser Permanente National Pharmacy, Aurora, CO USA; 3 Department of Clinical Pharmacy, Skaggs School of Pharmacy and Pharmaceutical Sciences, University of Colorado Anschutz Medical Campus, Aurora, CO USA; 4 Kaiser Permanente Southern California Infectious Diseases Clinic, Panorama City Specialty Medical Office, Panorama City, CA USA; 5 Kaiser Permanente Southern California Outpatient Infusion Pharmacy, Kaiser Permanente Panorama City Medical Center, 13652 Cantara St, Bldg 4, LL, Rm L21, Panorama City, CA 91402 USA; 6 Department of Research & Evaluation, Kaiser Permanente Southern California, Pasadena, CA USA

**Keywords:** Antimicrobial stewardship, Aminoglycosides, Quality improvement, Urinary tract infection

## Abstract

**Background:**

Urinary tract infections (UTI) are often over-diagnosed and over-treated, which can induce and select for resistant pathogens. After observing wide-spread outpatient use of ertapenem, a broad-spectrum antibiotic, a structured antimicrobial stewardship initiative (ASI) to improve appropriate antimicrobial prescribing was undertaken. ASI objectives were to achieve a goal of reducing ertapenem utilization for extended spectrum beta lactamase *Enterobacteriaceae* (ESBL-EB) UTI by 10% and evaluate the clinical outcomes associated with the ASI.

**Methods:**

A pre-to-post cohort study was conducted at a single-center integrated healthcare system between November 1, 2014 and February 26, 2017. An intensive, 90-day, pharmacist-driven, structured ASI was implemented between November 1, 2015 and January 29, 2016. Female patients aged ≥18 years who were treated for an uncomplicated, ESBL-EB urinary tract infection (UTI) were included. Primary outcome was clinical resolution defined as cure, persistence, relapse and recurrence. Secondary outcome measured was monthly ertapenem use expressed as number of days of therapy (DOT)/1000 adjusted patient days (APD). Segmented regression analysis for interrupted time series was performed to estimate ASI intervention effect.

**Results:**

A total of 184 patients were included in the study. Ertapenem utilization decreased from 0.0145 DOT/1000 APD in Nov. 2014 to 0.0078 DOT/1000 APD Feb. 2017(*p* < 0.01). The mean ertapenem DOT declined 19% overall from the pre vs. post intervention periods (32 vs 26, *p* < 0.01). Frequency of recurrent UTIs between treatments did not significantly differ and no adverse effects were reported in patients treated with aminoglycosides.

**Conclusions:**

A structured ASI for uncomplicated ESBL-EB UTI was associated with a clinically meaningful decrease in ertapenem utilization and once-daily, 5-day aminoglycoside treatment was well-tolerated.

## Background

Urinary tract infections (UTI) are often over-diagnosed and over-treated in a wide range of care settings [1, 2]. Misuse of broad-spectrum antimicrobials has been shown to select and induce resistant pathogens; as with the case of high emergence of extended spectrum B-lactamase *Enterobacteriaceae* (ESBL-EB) UTIs [3–5]. Therapeutic options for ESBL-producing organisms are limited because of concomitant resistance to other classes of antimicrobials. Ertapenem, a once daily broad-spectrum carbapenem, is often used as first line treatment [6]. Overuse of ertapenem, however, can subsequently lead to carbapenem resistance in a seemingly inescapable cycle [7].

Re-consideration of aminoglycoside therapy as a carbapenem sparing option for ESBL-EB UTIs is a particularly attractive treatment option due to its ability to achieve high peak-to-MIC ratio urine concentrations for prolonged periods, ease of administration, and relatively low toxicity [8, 9]. Furthermore, aminoglycoside therapy does not mediate resistance to other antimicrobial classes and is effective in single dose therapy and feasible as monotherapy for non-bacteremic ESBL-EB UTIs [10–12]. While limiting the use of an antimicrobial class through substitution has been a mainstay of antimicrobial stewardship efforts, national surveillance programs have identified that reduction in selective pressure is the key component of an effective strategy e.g., Delgado and colleagues’ retrospective study reported substantial reduction of ertapenem utilization while maintaining susceptibilities of *Pseudomonas aeruginosa* and Enterobacteriaceae [13].

Accelerating trends in overall ertapenem use and initial cases of carbapenem-resistant *Enterobacteriaceae* (CRE) were observed in a community medical center. A collaborative antimicrobial stewardship initiative (ASI), thus, was launched to improve antimicrobial utilization in the outpatient antibiotic parenteral therapy (OPAT) setting. Specifically, the scope of targeting treatment of uncomplicated ESBL-EB UTI was identified as a performance improvement opportunity to bridge the gap for evaluation and monitoring that was overlooked by inpatient antimicrobial stewardship programs [14]. The objective of the ASI was to achieve a SMART, (mnemonic acronym for **s**pecific, **m**easurable, **a**ttainable, **r**elevant, and **t**ime-bound criteria to guide in the setting of objectives) goal of reducing ertapenem utilization for ESBL-EB UTI by 10% in 90 days. Based on distribution fitting and concept of thresholds, 10% was selected as a performance goal with the general rule that 80% should be achievable, a target level should be 50% achievable, and a stretch goal should be about 20% achievable [15]. This study evaluated that goal and the clinical outcomes associated with the ASI. Findings from this study can provide insights on antibiotic prescribing in the OPAT setting.

## Methods

### Study Design & Setting

This retrospective, pre-to-post, cohort study evaluated female patients > 18 years of age with an uncomplicated ESBL-EB UTI in the OPAT setting who were dispensed a prescription for ertapenem or aminoglycoside between November 1, 2015 and February 26, 2017 at one medical center within Kaiser Permanente Southern California (KPSC), an integrated healthcare delivery system. The center includes a 218-bed tertiary hospital and five surrounding medical offices that provide primary and specialty care to 265,000 patients. Coded and free-text data on diagnoses, procedures, laboratory tests, medications, hospitalization, and membership are maintained in KPSC electronic medical record and pharmacy dispensing systems. All aspects of the study were reviewed and approved by the KPSC Institutional Review Board. Patient consent was not required due to the retrospective nature of the study.

At the time of the study, there were no internal protocols directing the use of aminoglycosides for ESBL-EB UTI. An ASI was designed to improve antimicrobial utilization through broad-based, pharmacy driven, and disease focused interventions. The ASI was led by the Chief of Infectious Diseases (ID) and an ID-trained outpatient pharmacist specialist, who collaborated closely with ordering providers (physicians and mid-level practitioners), laboratory, department administrators, and both inpatient and outpatient pharmacy. The study encompassed an ASI pre-implementation (September 1, 2014 - August 31, 2015), 90-day implementation period (November 1, 2015 - February 26, 2016), and bi-phasic post- implementation period (October 10, 2016 - February 26, 2017). A 2-month “wash-out” window between September 1, 2015 and October 31, 2015 was included to launch the initiative.

The index date for study inclusion was the date of UTI diagnosis defined as International Classification of Disease and Related Health Problems (ICD)-9 diagnosis- 595.5 and updated (ICD)-10) code for urinary tract infection (ICD-10 N30.0, ICD-9599.0) and related cystitis (ICD-10-N39.0). Patients were recruited via continuous enrollment in KPSC during the 6 months prior to study index date (required to collect baseline characteristics reliably). Each episode was counted as a unique episode if occurring > 30 days after the index date. Retrospective electronic chart review of all clinical documentation was performed to evaluate patient symptomatology, laboratory values, and to verify UTI diagnosis and outcomes. Infectious disease consultation/approval were required for all ESBL-EB UTI prescriptions. While fosfomycin and nitrofurantoin are recommended per Infectious Diseases Society of America (IDSA) 2010 guideline in the treatment of ESBL-EB UTIs, data collection for these oral options were excluded from the intervention as multiple barriers limit implementation in practice [6]. Several mechanisms of resistance resulting in heteroresistant subpopulations along with rates of treatment failure with fosfomycin ranging from 22 to 59% prevent routine use [16–19]. Additionally, fosfomycin susceptibility is not routinely included in testing for all confirmed ESBL-Enterobacteriaceae in most institutions. Similarly, treatment failure and adverse outcomes limit use of nitrofurantoin older patients with renal impairment [20]. Data collection focused on measurement of parenteral ertapenem and aminoglycoside allowed accurate measurement of the intervention’s core strategy.

Confirmation of diagnosis was made with a positive ESBL-EB urine culture of > 100,000 colony forming units (CFU) and were excluded from analysis if the culture had no/insufficient bacterial growth, normal/mixed flora, or classified by the microbiology laboratory as contamination on final report. Prior to 2018, ESBL was confirmed by disk method. In 2018, confirmation was discontinued as Clinical & Laboratory Standards Institute (CLSI) no longer recommends changing the interpretation of the beta-lactam based on detection of ESBL [21].

Outpatient setting was defined as an infusion center, urgent care department, emergency department, or home. 

Patients were excluded if they were not eligible for once daily dosing aminoglycoside therapy: pregnancy (as there are no data on fetal pharmacokinetics and toxicity), ascites (ICD-9- 789.59), endocarditis (ICD-9424.9X), dialysis, burns > 20% of body surface area, renal sufficiency (creatinine clearance (CrCl) ≥20 ml/min), bacteremia (determined by positive blood cultures), hemodynamic instability, and immunocompromised neutropenia (absolute neutrophil count < 1500/μl). In addition, patients with other urinary tract-related infections, pyelonephritis, or a complicated UTI (defined as UTI occurring in male or association with a structural or functional abnormality of the genitourinary tract including existing kidney stones or preexisting conditions involving the urinary tract) were excluded [22].

### Intervention

The intervention period (November 1, 2015 - February 26, 2016) was marked by educational presentations including formal seminars by the ID chief and Post Graduate Year (PGY)-1 pharmacy resident to obtain buy-in from multiple partners. Medical specialties targeted for education included hospitalist, emergency medicine/urgent care physicians, intensivists, and pharmacists. Using an inter-disciplinary multi-level approach based on Society for Healthcare Epidemiology of America (SHEA) antimicrobial stewardship guidelines [22], the core strategy was to provide a prospective audit to prescribers and tighten adherence to formulary restriction and pre-authorization for ertapenem use for ESBL UTIs. This was spearheaded by ID physicians and outpatient infusion pharmacists through one-on-one, prescriber-level, patient-specific antimicrobial “time-outs” to explore the need for treatment and consideration of once-daily aminoglycosides as first-line therapy where appropriate. In addition, specialty detailing of antimicrobial use was performed in a stepwise transition: first with medical offices and outlying clinics followed by emergency department and urgent care sites.

**Fig. 1:**
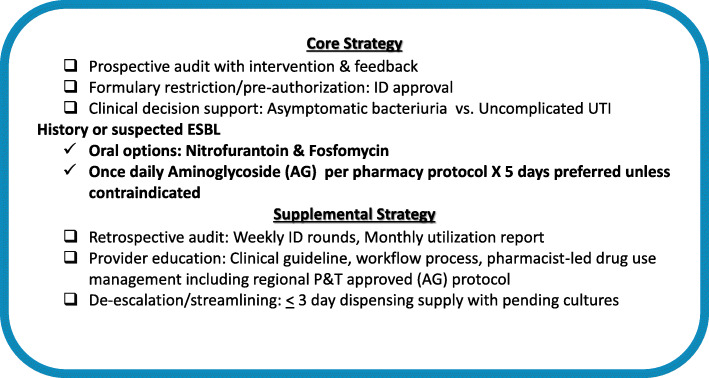
Outpatient Infusion Pharmacy Antimicrobial Stewardship Initiative

Supplemental strategies to educate providers included guideline navigation, non-treatment of asymptomatic bacteriuria, routine restriction of fluoroquinolone and third generation cephalosporin for cystitis, upstream limiting of duration of therapy (i.e., from seven to 5 days), approval from regional pharmacy and therapeutics committees for outpatient collaborative management of aminoglycoside, and subsequent development of work flow process for referral to “per pharmacy protocol” (Table 1) [23, 24]. De-escalation/streamlining was performed by limiting empiric ertapenem dispensing supply to < 3 days with pending cultures and without previous history of ESBL bacteria or significant risk factors (i.e., previous use of broad-spectrum antibiotics, hospitalization within 90 days, history of recurrent UTI, or presence of urological abnormalities) [25, 26]. Retrospective audit through weekly ID rounds and monthly utilization reporting provided feedback on antimicrobial use and patient outcomes (Fig. 1). The iterative control cycle Plan-Do-Study-Act performance improvement method was used to identify threshold drop-off for sustainability.

**Table 1:** Outpatient Infusion Pharmacy Services Managed Protocol

**Referring provider**	Determine the need for Aminoglycoside therapy, duration of therapy and consult with ID physician if applicable or required at the local medical center.
**Authorized Pharmacist Functions**:	Gather Patient-Specific Information. Order and evaluate appropriate laboratory work, Initiate aminoglycoside therapy using accepted adult dosing guidelines
Demographics	Age, Gender, Height, and Actual Body Weight (ABW)
Labs	Serum Creatinine (SCr), Blood Urea Nitrogen (BUN), Complete Blood Count (CBC), Cultures & Sensitivities
History	Allergies Medical and Medication History Diagnosis/Reason for Vancomycin Previous aminoglycoside pharmacokinetic data
Order and evaluate appropriate lab work	Baseline SCr Routine CBC with differential, SCr +/− BUN, and aminoglycoside trough weekly or more frequently based on patient’s age, history, concurrent nephrotoxic or ototoxic medications and clinical judgment. May order peak level per clinical judgment or patient case. Trough level should be done prior to 3rd to 5th dose after initiation, change in dosage and at least once a week if creatinine is stable, and more often for changes in creatinine of greater than or equal to 0.5 mg/dL. If there is a greater than or equal to 0.5 mg/dL change in serum creatinine, a trough and/or repeat random level should be done with the next feasible dose.
**Single Daily Dose Aminoglycoside (SDDA)** Gentamicin/Tobramycin 5 mg/kg/dose IV Amikacin 15 mg/kg/dose IV	Exclusion i. Pregnant women (no data on fetal pharmacokinetics and toxicity) ii. Ascites iii. Endocarditis iv. Dialysis patients v. Burn patients vi. Patients with ESRD or with CrCl < 20 mL/min vii. Patients who are hemodynamically unstable viii. Neutropenic or critically ill patients

### Outcomes

This study described patient demographics, prescription characteristics, and organisms observed on urine cultures in the 90 days pre and post diagnosis index date. The primary outcome was clinical resolution defined as cure (complete resolution of signs and symptoms of infection e.g. dysuria, urinary frequency/urgency, suprapubic pain, and or fever), persistence (same bacteria cultured in the urine 2 weeks after initiating sensitivity-adjusted therapy) relapse (recurrence with the same or different organism > 2 weeks after treatment or a sterile intervening culture. Recurrent was defined as > 2 UTIs in the last 6 months or 3 or more UTIs in the last 12 months or readmission for UTI with 90-day re-treatment [6]. Antibiotic susceptibility was based on Vitek 2 (bioMerieux, Durham, NC) technology. Interpretive criteria used by the microbiology laboratory was based on Clinical Laboratory Standards Institute document M100. Safety evaluations included documented adverse effects and or acute kidney injury (AKI) defined as > 50% increase in serum creatinine levels within < 7 days.

Secondary outcomes measured were ertapenem utilization defined as number of days of therapy (DOT)/1000 adjusted patient days (APD). The use of DOT/1000 APD is currently the most accurate and preferred measure of antibiotic use and is used by the Centers of Disease Control and Prevention (CDC) and National Healthcare Safety Network (NHSN). This metric allows for comparison of antibiotic utilization both within and between institutions and services of different sizes when normalized to patient days. It is unaffected by change in dosing [27].

### Data collection

Baseline information on patient characteristics, facility level antimicrobial susceptibility patterns, antimicrobial utilization, and clinical outcomes were collected from the OPAT administration database and electronic medical record system. Patients were assessed for comorbidities, initial symptoms and characteristics of UTIs (upper or lower, complicated or uncomplicated), and improvement of symptoms. Laboratory data included serum creatinine (as surrogate marker for renal function before and after therapy), anatomical or functional abnormalities, urine culture positive for ESBL-EB, appropriateness of treatment based on selection of antimicrobial, dose, duration, and eligibility for aminoglycoside or oral alternative using a validated data collection template [25].

Aminoglycoside dose and dosing interval were determined by body weight and renal function and adjusted per collaborative practice agreement. Retrospective electronic chart reviews were conducted by multiple un-blinded pharmacist reviewers under the supervision of site investigators.

### Data analysis

Summary statistics were described for categorical variables and continuous variables. The comparison of aminoglycoside prescription rates was performed with the Chi-square test of association. The monthly trend of aminoglycoside prescription rates after intervention was evaluated by Cochran-Armitage test. Comparison by t-test of the mean ertapenem therapy days per total days of therapy between the pre- and post ASI initiation time periods was conducted. Segmented regression analysis for interrupted time series was used to determine the significance of the differences in levels and slopes of ertapenem utilization over time after implementation of the two intervention phases. Estimations were made of the change in ertapenem utilization, the difference between the pre- and post-intervention slopes of the outcome, and the 12-month intervention effect after the intervention. Significance was defined as *p* < 0.05. All statistical analyses were performed with SAS version 9.4 (SAS Institute Inc., Cary, NC).

## Results

### Patients

**Fig. 2:**
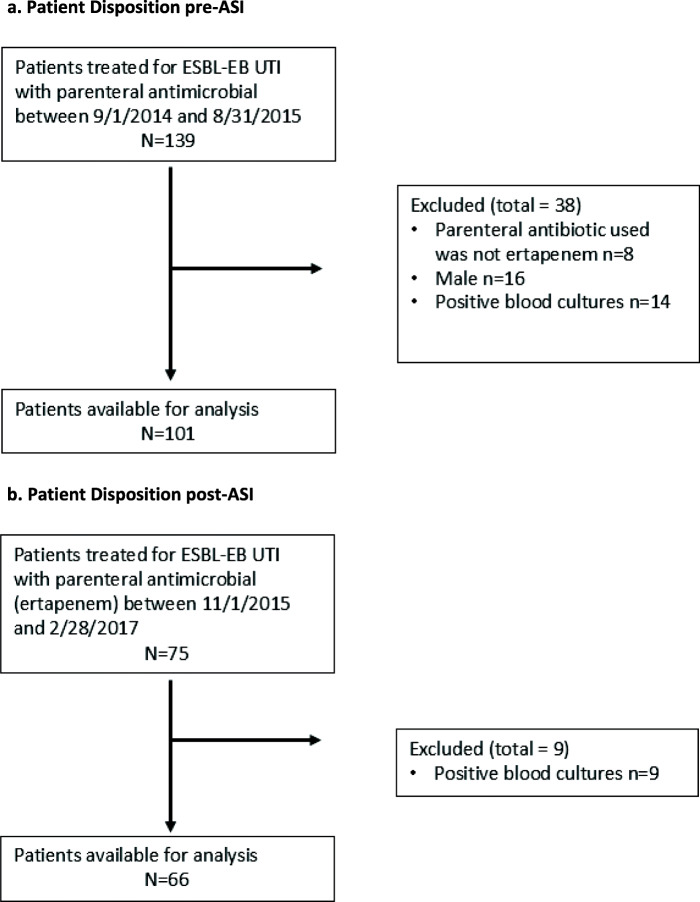
**a** Patient Disposition pre-ASI. **b** Patient Disposition post-ASI

A total of 184 patients treated outpatient for ESBL UTI with either ertapenem or aminoglycosides were evaluated. During the pre-intervention period (September 1, 2014- August 31, 2015), a total of 139 patients were reviewed and 101 were identified as having been treated for ESBL-EB UTI with ertapenem who could have been appropriately treated with once-daily aminoglycoside treatment (Fig. 2a). Baseline characteristics were similar between the two groups (Table 2). The mean age was 67 + 17 years and mean renal function as expressed by Creatinine clearance (mL/min) was 61.7 + 34. The average temperature and white blood cell counts were within normal range. Most patients had pyuria and positive markers of urinary tract inflammation on urinalysis. Gram negative rods predominated positive urine cultures for *Escherichia coli* (70%) with 58% of isolates being ESBL *E. coli*. Susceptibility to aminoglycoside accounted for 82% of cultures. Of parenteral prescriptions reviewed, ertapenem had been prescribed 100%.

**Table 2:** Patient Characteristics by Intervention Status (*N* = 183)

Characteristic	Pre-intervention Group (9/1/14 to 8/31/15) *n* = 101	Post-intervention Group^**a**^ (11/2/15 to 2/28/17) *n* = 83	***P***-value
Mean age (years+/− SD)	67.5 ± 16.9	68.9 ± 19.0	0.45
Mean creatinine clearance (+/− SD)	61.7 ± 34.5	59.7 ± 27.3	0.97
Creatinine clearance, n (%)			0.73
≥ 20 mL/min	95 (94)	79 (96)	
< 20 mL/min	6 (6)	3 (4)	
Positive for UTI symptoms, n (%)	84 (83)	74 (89)	0.25
Positive urine culture, n (%)	91 (90)	72 (87)	0.48
Cultured organism, n (%)			0.08
E.coli	70 (69)	75 (90)	
ESBL E.coli	59 (58)	46 (56)	
Contamination	6 (6)	1 (1)	
Other	15 (15)	11 (13)	
None	6 (6)	1 (1)	
Prior culture results used for Treatment n (%)	4 (4)	5 (6)	
Culture sensitive to aminoglycoside, n (%)			0.46
Amikacin	38 (38)	37 (45)	
Gentamicin	45 (44)	36 (43)	
Unable to assess	18 (18)	10 (12)	
Colony counts > 100 k, n (%)	77 (76)	66 (80)	0.59

^a^ Treated with ertapenem or aminoglycoside

During the ASI intervention and follow up period (November 1, 2015- February 28, 2017), a total of 75 patients were reviewed and 66 were identified as having been treated for ESBL-EB UTI with ertapenem who also could have been appropriately treated with once-daily aminoglycoside (Fig. 2b). Seventeen patients were identified as having been treated for ESBL UTI with amikacin. Mean age was 68.86 ± 19.03 years and mean renal function as expressed by Creatinine clearance (mL/min) was 59.66 ± 27.26. Susceptibility to aminoglycoside accounted for 85% of cultures taken from the patients treated with ertapenem.

### Primary outcome

Recurrent urinary tract infection did not differ significantly (*p* = 0.57) occurring in 46 patients (28%) treated with ertapenem and 3 patients (18%) treated with aminoglycosides (Table 3). Adverse effects reported in 5 patients receiving ertapenem were nausea, vomiting, bilateral extremity heaviness, facial swelling, cramping, hallucination, rash/petechiae and intestinal cramping and loss of appetite. No adverse effects or acute kidney injury were reported in patients receiving aminoglycosides. There was no significant difference found between rate of adverse effects found in either treatment group (*p* = 0.99) (Table 3).

**Table 3:** Efficacy and Safety

Efficacy and Safety	Ertapenem *N* = 167	Aminoglycoside *N* = 17	***P***-value
**Efficacy**			
Return to hospital or clinic within 90 days for UTI, n (%)	46 (28)	3 (18)	0.57
**Safety**			
Any Adverse effect	5 (3) Nausea/vomiting, bilateral extremity heaviness, facial swelling, cramping, hallucination, rash/petechiae and intestinal cramping and loss of appetite	0 (0) No incidence of ototoxicity or nephrotoxicity	0.99

### Assessment of ASI impact

**Fig. 3:**
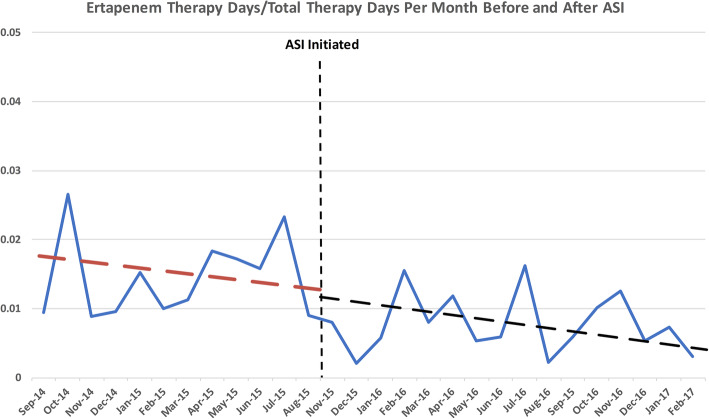
Ertapenem Use Over Time

Figure 3 depicts the trend of monthly ertapenem DOT before and after ASI implementation. The overall trend line is statistically significantly reducing over time (*p* < 0.01). Linear modeling of the trend lines pre- and post-ASI initiation (i.e., interrupted time series) shows the post-period trend is statistically significantly different than the pre-period trend (*p* < 0.01) for the interaction between period and month with a change in trend of < 2%/month).

### Secondary outcome

Ertapenem utilization decreased from 0.0145 DOT/1000 APD in Nov. 2014 to 0.0078 Dot/1000 APD Feb. 2017 (*p* < 0.01). The mean monthly ertapenem DOT declined 19% overall from the pre vs. post intervention periods (32 vs 26, *p* < 0.01).

## Discussion

This retrospective assessment of an ASI for antimicrobial utilization for UTI in the OPAT setting identified that the ASI was associated with a sustained ertapenem utilization decrease from the pre- to post-ASI intervention periods and an overall mean ertapenem DOT decline of 19%. In addition, there was no difference in the rates of return for additional treatment or recurrent UTI between the study periods. These findings support that an ASI that prioritizes use of an aminoglycoside over ertapenem can be implemented without adverse clinical consequences.

While our disease-focused ASI succeeded in exceeding the a priori SMART goal of 10% reduction in ertapenem utilization for ESBL-EB UTI by driving conversion to non-carbapenem alternatives, its sustainability relied on a multi-faceted strategy. Most surprising of this intervention was the prolonged acceptance of recommendations for reduction of duration of therapy for ESBL-EB UTI from 7 days to 5 days. In addition, while drop-off was anticipated after the intense ASI initiative, loss of continued active engagement through prospective audits to prescribers and pre-authorization by OPAT pharmacist and infectious disease physician champions required sustained efforts. This finding corroborates that extension of antimicrobial stewardship to the ambulatory setting can be as effective as other settings of care [28]. Nevertheless, persistent active efforts through feedback to clinicians are required in order to sustain initial improvements in prescribing [29].

One strength of our study is the inclusion of a strict definition of UTI that included only uncomplicated ESBL-EB UTIs with negative blood cultures. This allowed for once daily aminoglycoside monotherapy so it would be comparable to ertapenem administration. Zohar and colleagues recently demonstrated the efficacy and safety of aminoglycosides in treating blood-stream infections of urinary source caused by ESBL-EB [30]. This carbapenem-sparing approach suggests an opportunity to expand aminoglycoside monotherapy conversion to complicated ESBL-EB UTIs with the potential for decreased admissions hospitalization and duration of hospitalization.

Another strength of this study was its real-world context. While time series analysis ruled out temporal trends and other confounding factors such as new staff/emergency department gap in education and prescribing patterns, deliberate collaborative planning was essential for identification of barriers and closure of practice gaps. We identified barriers to implementation including lack of uniform OPAT collaborative practice agreement for aminoglycoside management, preliminary weak alignment of hospital pharmacy and OPAT treatment of ESBL-EB UTIs, and lack of point of ordering clinical decision support. Pre-identified leadership support expedited consensus for regional approval of OPAT collaborative practice for aminoglycoside management. Formal education, peer to peer communication, and peer benchmarking by ASI champions provided performance incentivization. Of note, while the 2020 IDSA Focus on Extended Spectrum B-Lactamase Producing Enterobacterales Guideline was released after our pilot, our practice is consistent with those guidelines. Options such as amoxicillin-clavulanate, single intravenous dose aminoglycoside, and oral fosfomycin were considered. They did not comprise the backbone of the ASI because of association of higher rates of clinical failure or lower level of evidence supporting use [24]. Lastly, findings from this study provided proof of concept to advance the case for innovation of outpatient electronic referral work-flow processes.

Future application of the ASI would be to integrate a model of care that optimizes OPAT pharmacist workflow to jointly provide prospective clinical decision support with infectious disease physicians for ordering providers. It could mirror established hospital antimicrobial stewardship programs to reduce overall antimicrobial usage to even include evaluation of oral high-risk broad-spectrum antimicrobials [31]. Another next step for aligning system level performance improvement would be to enhance electronic technology capabilities to incorporate bundled electronic smart order sets, allow for same time feedback, and automate reporting functions.

Our study had several limitations. We were unable to measure patient satisfaction with care. Since aminoglycosides require routine monitoring, patients had to return to the laboratory or receive a phlebotomist visit for a blood draw. Institutional costs and patient burden associated with blood draws were not measured. In evaluating the local medical center antibiogram data from 2014 to 2020, susceptibility to trimethoprim-sulfamethoxazole was 74% for urinary isolates of *E. coli* and data was not available for susceptibility to Amoxicillin-clavulanate for urinary isolates. Susceptibility of E.coli from blood and other sources to Ampicillin/sulbactam ranged from 51 to 56%. *Escherichia coli* susceptibility, including that of ESBL-producing isolates, remained relatively consistent and no increase in aminoglycoside-resistant *Pseudomonas aeruginosa* was seen. Additionally, we were unable to assess rates of *Clostridium difficile* due to the unavailability of local data. These data were only available at the KPSC regional antibiogram level. Thus, the full consequence of antimicrobial pressure on clinical cultures of these resistant organisms could not be determined. Lastly, we did not perform medication cost analyses. It is estimated that aminoglycosides were far less expensive than ertapenem.

## Conclusions

Once-daily aminoglycosides with 5-day duration of therapy conversion from ertapenem combined in a collaborative inter-disciplinary multi-faceted ASI to include reduction of duration of therapy and active interventions appears to be a safe and effective route for treatment of uncomplicated ESBL UTI. This benchmark ASI proactively recognized the need for outpatient antimicrobial stewardship by developing a framework to address the Joint Commission’s expanded requirement for outpatient facilities to have antimicrobial stewardship programs to maintain accreditation effective January 2020 [31]. Future studies should evaluate the ASI in a prospective manner.

## Data Availability

Individual level data may not be made publicly available due to IRB and privacy concerns. The data used for this study contain protected health information (PHI) and access is protected by the Kaiser Permanente Southern California IRB. Data are available for researchers who meet the criteria for access to confidential data. For more information about data access and criteria for access to confidential data, please contact Isabel.M.Sanch@kp.org. All other relevant data are within the paper.

## Abbreviations

UTI: Urinary tract infection ASI Antimicrobial stewardship initiative
ESBL-EB: Extended spectrum B-lactamase *Enterobacteriaceae*
CRE: Carbapenem-resistant *Enterobacteriaceae*
OPAT: Outpatient antibiotic parenteral therapy
KPSC: Kaiser Permanente Southern California
SMART: **S**pecific, **m**easurable, **a**ttainable, **r**elevant, and **t**ime-bound
ID: Infectious Disease
ICD: International Classification of Disease and Related Health Problems
IDSA: Infectious Diseases Society of America
CFU: Colony forming units
CrCl: Creatinine clearance
SHEA: Society for Healthcare Epidemiology of America
DOT: Days of therapy
APD: Adjusted patient days
CDC: Centers of Disease Control and Prevention
NHSN: National Healthcare Safety Network

## References

[1] Zalmanovici Trestioreanu A, Lador A, Sauerbrun-Cutler MT, Leibovici L (2015). Antibiotics for asymptomatic bacteriuria. Cochrane Database Syst Rev.

[2] Goto T, Yoshida K, Tsugawa Y, Camargo CA, Hasegawa K (2016). Infectious disease-related emergency department visits of elderly adults in the United States, 2011-2012. J Am Geriatr Soc.

[3] Cantas L, Suer K, Guler E, Imir T (2016). High Emergence of ESBL-Producing E coli cystitis: time to get smarter in Cyprus. Front Microbiol.

[4] Fernando MM, Luke WA, Miththinda JK, Wickramasinghe RD, Sebastiampillai BS, Gunathilake MP (2017). Extended spectrum beta lactamase producing organisms causing urinary tract infections in Sri Lanka and their antibiotic susceptibility pattern -a hospital based cross sectional study. BMC Infect Dis.

[5] Lim CL, Lee W, Lee AL, Liew LT, Nah SC, Wan CN (2013). Evaluation of Ertapenem use with impact assessment on extended-spectrum beta-lactamases (ESBL) production and gram-negative resistance in Singapore General Hospital (SGH). BMC Infect Dis.

[6] Gupta K, Hooton TM, Naber KG, Wullt B, Colgan R, Miller LG, Moran GJ, Nicolle LE, Raz R, Schaeffer AJ, Soper DE, Infectious Diseases Society of America, European Society for Microbiology and Infectious Diseases. Infectious Diseases Society of America; European Society for Microbiology and Infectious Diseases (2011). International clinical practice guidelines for the treatment of acute uncomplicated cystitis and pyelonephritis in women: a 2010 update by the Infectious Diseases Society of America and the European Society for Microbiology and Infectious Diseases. Clin Infect Dis.

[7] Durante-Mangoni E, Andini R, Zampino R (2019). Management of carbapenem-resistant *Enterobacteriaceae* infections. Clin Microbiol Infect.

[8] Tulara NK (2018). Nitrofurantoin and Fosfomycin for extended Spectrum Beta-lactamases producing *Escherichia coli* and *Klebsiella pneumoniae*. J Glob Infect Dis.

[9] Derington CG, Benavides N, Delate T, Fish DN (2020). Multiple-dose oral fosfomycin for treatment of complicated urinary tract infections in the outpatient setting. Open Forum Infect Dis.

[10] Goodlet KJ, Benhalima FZ, Nailor MD (2018). A systematic review of single-dose aminoglycoside therapy for urinary tract infection: is it time to resurrect an old strategy?. Antimicrob Agents Chemother.

[11] Cho SY, Choi SM, Park SH, Lee DG, Choi JH, Yoo JH (2016). Amikacin therapy for urinary tract infections caused by extended-spectrum β-lactamase-producing Escherichia coli. Korean J Intern Med.

[12] Vidal L, Gafter-Gvili A, Borok S, Fraser A, Leibovici L, Paul M (2007). Efficacy and safety of aminoglycoside monotherapy: systematic review and meta-analysis of randomized controlled trials. J Antimicrob Chemother.

[13] Delgado A, Lee GC, Gawrys GW, Duhon BM, Koeller JM (2015). Impact of an antimicrobial stewardship initiative on ertapenem use and carbapenem susceptibilities at four community hospitals. Open Forum Infectious Diseases.

[14] Morris AM, Bai A, Burry L, Dresser LD, Ferguson ND, Lapinsky SE, Lazar NM, McIntyre M, Matelski J, Minnema B, Mok K, Nelson S, Poutanen SM, Singh JM, So M, Steinberg M, Bell CM (2019). Long-term effects of phased implementation of antimicrobial stewardship in academic ICUs: 2007-2015. Crit Care Med.

[15] Courtlandt CD, Noonan L, Field LG (2009). Model for improvement- part1: a framework for healthcare quality. Pediatr Clin North Am.

[16] Portillo-Calderón I, Ortiz-Padilla M, Rodríguez-Martínez JM, de Gregorio-Iaria B, Blázquez J, Rodríguez-Baño J, Pascual A, Docobo-Pérez F (2020). Contribution of hypermutation to fosfomycin heteroresistance in Escherichia coli. J Antimicrob Chemother.

[17] Doesschate T, Abbott IJ, Willems RJL, Top J, Rogers MRC, Bonten MM (2019). *In vivo* acquisition of fosfomycin resistance in *Escherichia coli* by *fosA* transmission from commensal flora. J Antimicrob Chemother.

[18] Hatlen TJ, Flor R, Nguyen MH, Lee GH, Miller LG (2020). Oral fosfomycin use for pyelonephritis and complicated urinary tract infections: a 1 year review of outcomes and prescribing habits in a large municipal healthcare system. J Antimicrob Chemother.

[19] Neuner EA, Sekeres J, Hall GS, van Duin D (2012). Experience with fosfomycin for treatment of urinary tract infections due to multidrug-resistant organisms. Antimicrob Agents Chemother.

[20] The American Geriatrics Society 2015 Beers Criteria Update Expert Panel (2015). American Geriatric Society 2015 updated beers criteria for potentially inappropriate medication use in older adults. J Am Geriatr Soc.

[21] CLSI Performance Standards for Antimicrobial Susceptibility Testing. 30^th^ ed. CLSI Guideline M100. Wayne: Clinical and Laboratory Standards Institute; 2020.

[22] Barlam TF, Cosgrove SE, Abbo LM, MacDougall C, Schuetz AN, Septimus EJ, Srinivasan A, Dellit TH, Falck-Ytter YT, Fishman NO, Hamilton CW, Jenkins TC, Lipsett PA, Malani PN, May LS, Moran GJ, Neuhauser MM, Newland JG, Ohl CA, Samore MH, Seo SK, Trivedi KK (2016). Implementing an antibiotic stewardship program: guidelines by the Infectious Diseases Society of America and the Society for Healthcare Epidemiology of America. Clin Infect Dis.

[23] Nicolle LE, Gupta K, Bradley SF, Colgan R, DeMuri GP, Drekonja D, Eckert LO, Geerlings SE, Köves B, Hooton TM, Juthani-Mehta M, Knight SL, Saint S, Schaeffer AJ, Trautner B, Wullt B, Siemieniuk R (2019). Clinical practice guideline for the management of asymptomatic bacteriuria: 2019 update by the Infectious Diseases Society of America. Clin Infect Dis.

[24] Tamma P, Aitken SL, Bonomoa RA, Mathers AJ, Van Duin D, Clancy CJ. Infectious Diseases Society of America guidance on the treatment of antimicrobial resistant gram-negative infections. Infectious Diseases Society of America 2020; Available at https://www.idsociety.org/practice-guideline/amr-guidance/. 10.1093/cid/ciad428 37463564

[25] CDC Assessment of Appropriateness of Antibiotics for Urinary Tract Infection. https://www.cdc.gov/antibiotic-use/healthcare/pdfs/UTI-Assessment.docx. Accessed 20 Dec 2019.

[26] Thakkar K, Gilchrist M, Dickinson E, Benn J, Franklin BD, Jacklin A, Anti-infective Policy Implementation Group (2011). A quality improvement programme to increase compliance with an anti-infective prescribing policy. J Antimicrob Chemother.

[27] Ibrahim OM, Polk RE (2014). Antimicrobial use metrics and benchmarking to improve stewardship outcomes. Methodology, opportunities, and challenges. Infect Dis Clin N Am.

[28] Gerber JS, Prasad PA, Fiks AG, Localio AR, Bell LM, Keren R, Zaoutis TE (2014). Durability of benefits of an outpatient antimicrobial stewardship intervention after discontinuation of audit and feedback. JAMA..

[29] Doernberg SB, Dudas V, Trivedi KK (2015). Implementation of an antimicrobial stewardship program targeting residents with urinary tract infections in three community long-term care facilities: a quasi-experimental study using time-series analysis. Antimicrob Resist Infect Control.

[30] Zohar I, Schwartz O, Yossepowitch O, David SSB, Maor Y (2020). Aminoglycoside versus carbapenem or piperacillin/tazobactam treatment for bloodstream infections of urinary source caused by gram-negative ESBL-producing Enterobacteriaceae. J Antimicrob Chemother.

[31] Joint Commission (2020). New antimicrobial stewardship in ambulatory health care standard.

